# Video-assisted thoracoscopic segmentectomy with combined chest wall resection: A case report

**DOI:** 10.1186/s13019-022-01996-3

**Published:** 2022-10-10

**Authors:** Yoichi Ohtaki, Toshiki Yajima, Kai Obayashi, Seshiru Nakazawa, Hayato Ikota, Ken Shirabe

**Affiliations:** 1 Department of General Surgical Science, Gunma University Graduate School of Medicine, Maebashi, Gunma Japan; 2 Department of Innovative Cancer Immunotherapy, Gunma University Graduate school of Medicine, Maebashi, Gunma Japan; 3 Clinical Department of Pathology, Gunma University Hospital, Maebashi, Gunma Japan

**Keywords:** Lung cancer, Video-assisted thoracoscopic surgery, Segmentectomy, Chest wall

## Abstract

**Background:**

Resection of lung cancer with chest wall involvement is an invasive procedure.

**Case presentation:**

We report a case of pulmonary adenocarcinoma with chest wall involvement that was resected through video-assisted thoracoscopic segmentectomy and combined en bloc resection of the chest wall (2nd to 4th ribs). Surgical stress was decreased by reducing the extent of lung parenchymal resection and applying a video-assisted technique with an additional posterior paravertebral incision.

**Conclusion:**

A thoracoscopic surgical approach involving incisions in areas requiring resection of the proximal, lateral, and posterior sides of the involved ribs can be applied to tumors invading the chest wall.

## Background

Video-assisted thoracoscopic surgery (VATS) is a minimally invasive approach for early-stage non-small-cell lung cancer (NSCLC). Minimally invasive approaches reduce surgical stress, consequently shortening the length of hospitalization and ensuring a better patient quality of life (1). However, reports on the use of VATS for lung cancer requiring the combined resection of multiple ribs are limited. Thus, we present a case of lung cancer with chest wall invasion that was managed by VATS segmentectomy with en bloc resection of three ribs.

## Case presentation

**Fig. 1:**
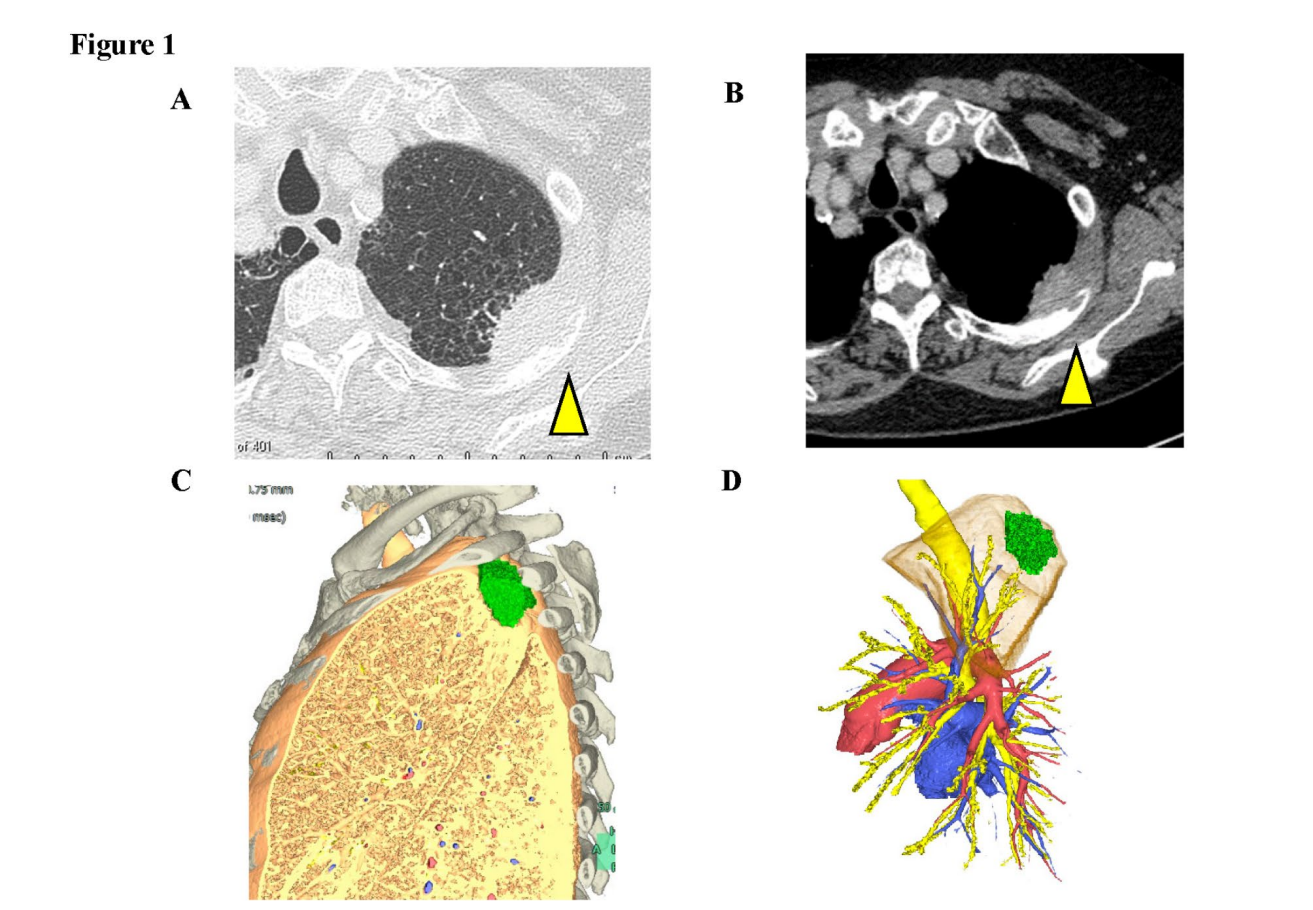
**Radiographic findings.** Chest computed tomography revealed a 4.5-cm mass with osteolytic changes in the adjacent third rib (**A, B**). Three-dimensional images showing the tumor location at the apicoposterior segment between the second and fourth ribs (**C, D**)

A 69-year-old woman experiencing back pain on her left side was referred to our hospital. Chest computed tomography (CT) revealed a 4.5 × 3.8 cm mass in the apicoposterior segment of the left upper lobe, invading the third rib (Fig. 1). Mediastinal lymphadenopathy was not observed. Pathological examination with CT-guided needle biopsy revealed lung adenocarcinoma. No evidence of distant metastasis was found on F-18 fluorodeoxyglucose positron emission tomography and brain magnetic resonance imaging. The tumor was diagnosed as a cT3N0M0 stage IIB lung adenocarcinoma. Although forced expiratory volume in one second (FEV1.0) and forced vital capacity (FVC) were almost normal (FEV1.0: 2290ml, 69.8%, FVC: 3280ml, 122.8%), the diffusing capacity of the lung for carbon monoxide (DLCO) was low (42.5%). While lobectomy is the first choice when the respiratory function is not considered, segmentectomy (an apicoposterior segmentectomy) with combined resection of the chest wall was selected in the present case to preserve the pulmonary function. The predicted postoperative %DLCO was 40.1%.

**Fig. 2:**
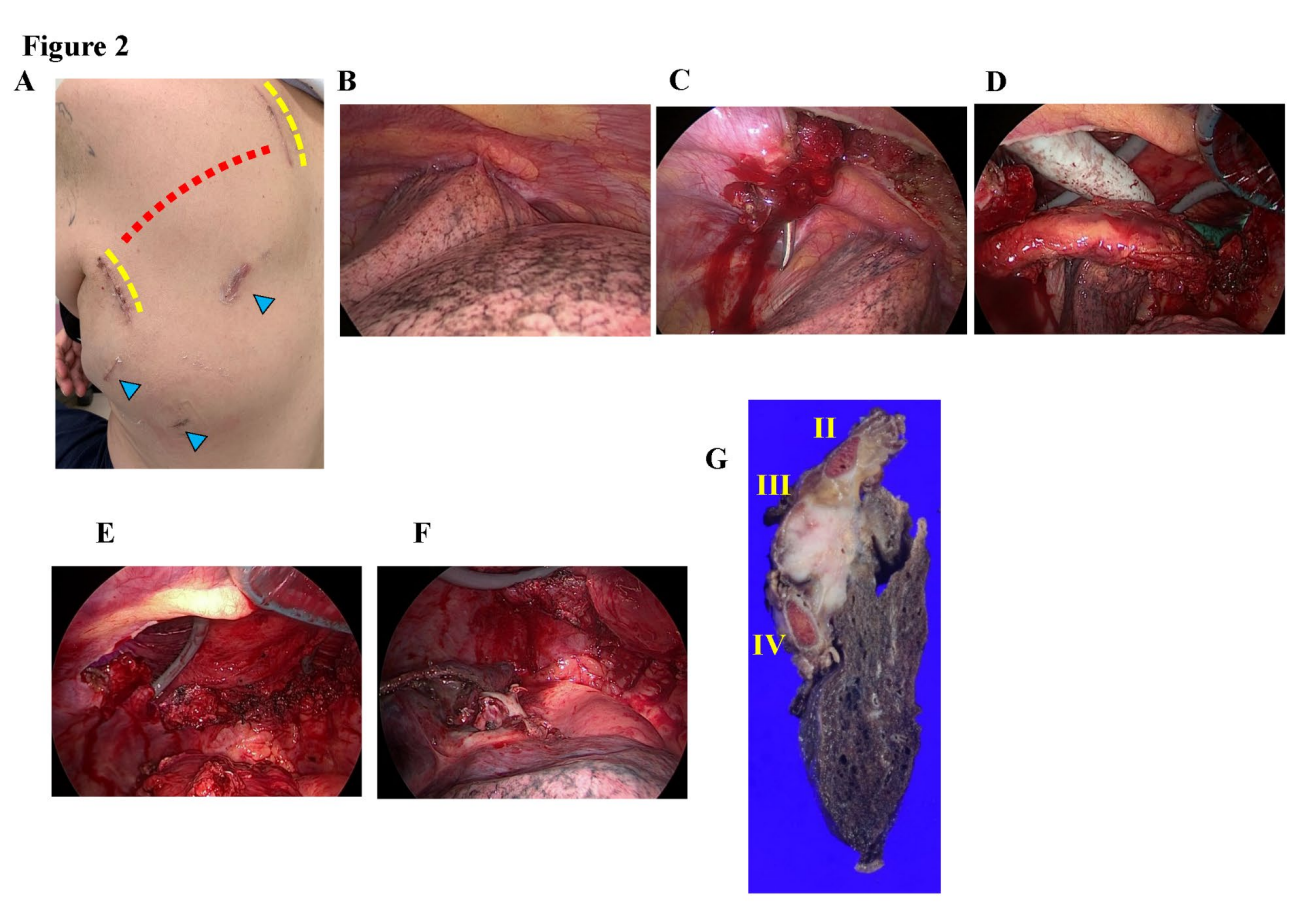
**Surgical findings.** Skin incisions (**A**). Three ports (blue arrowhead) and two access windows were used to resect the tumor. To bridge the anterior and posterior edges of the ribs, two 6-cm-sized windows (yellow dotted lines) were placed across the ribs (red dotted line) Intrathoracic findings (**B**). The chest wall was resected first with manual manipulation from two access windows (**C**–**E**). The left apicoposterior segmentectomy was then completed (**F**). Pathological findings show adenocarcinoma with invasion up to the third rib (**G**)

Although a four-incision approach was selected based on our standard surgical technique, the access port site was modified to allow resection of the anterior parts of the 2nd to 4th ribs (Fig. 2 A). Initial resection of the chest wall was performed because of the difficulty in manipulating hilar structures. Intraoperative findings revealed tumor invasion of the chest wall from the 2nd to 4th ribs (Fig. 2B). Resection of the chest wall began from the ventral and caudal sides of the ribs (Fig. 2 C). An additional 6-cm posterior incision along the left paravertebral line was made to allow resection of the ribs at the vertebral side, and the trapezius and rhomboid muscles were split. The tumor was manually operated on from both the ventral and paravertebral sides, and the ribs were resected with sufficient surgical margins (Fig. 2D). The resection was completed through the resection of the chest wall up to the level of the second rib on the cranial side (Fig. 2E). The resected ribs were attached to the left upper lobe, and a left apicoposterior segmentectomy with systematic lymph node dissection was performed en bloc with the ribs (Fig. 2 F). We used selective jet ventilation and intravenous indocyanine green injection to identify the intersegmental border, as described previously. 2 Because emphysematous changes were observed, resection segmental planes were created with staplers. The resected lung tissue was extracted using an anterior access window.

Chest wall reconstruction was not performed because the resected portion was completely covered by the scapula even upon elevation of the left arm. The operative time and blood loss were 370 min and 282 mL, respectively.

The postoperative course was uneventful and the patient was discharged on the 5th postoperative day. The final pathological finding was pT3N0M0 solid adenocarcinoma with invasion of only the third rib (Fig. 2G). Adjuvant cytotoxic chemotherapy was not administered due to patient refusal. No evidence of recurrence was observed 1 year post-surgery.

## Discussion and conclusions

Lobectomy with combined resection is the standard surgical procedure for NSCLC showing invasion of the adjacent organs. Minimally invasive techniques for lung cancer with chest wall invasion have been reported to reduce surgical stress (3–7). Cerfolio et al. first reported a “minimally invasive chest wall resection,” which eliminated cutting of the extrathoracic (trapezius, rhomboid, and serratus anterior) muscles (4). A hybrid VATS approach was initially applied to this technique (3), followed by the complete VATS approach (6, 7). Previous reports suggested resection of up to two ribs through a complete VATS approach (6, 7). However, no previous cases have reported the resection of three ribs together.

VATS segmentectomy is performed to reduce invasiveness and avoid overall complications. Consequently, the patient quality of life is improved and the hospital stay is decreased (1). In patients with NSCLC with chest wall invasion, sufficient surgical margins must be secured. Through modification of port placement, we were able to apply the VATS approach even for a tumor that required wide resection of the chest wall. Although the number of skin incisions could have been reduced, this method has the advantage of using both visualization and palpation to ensure adequate surgical margins on both ventral and dorsal sides.

As described by Dal Agnol et al., reconstruction of the chest wall was not performed because the tumor was located posteriorly and above the 5th rib; thus, the chest wall defect was covered and protected by the scapula and overlying muscles (5). For tumors widely invading the dorsal part of the central ribs, this technique may not be applicable. However, this procedure may be applied even in cases requiring chest wall reconstruction.

In conclusion, VATS pulmonary resection combined with chest wall resection can be achieved through the placement of incisions that bridge the resected ribs. The VATS approach is useful for visual and palpatory recognition of the surgical margins.

## Data Availability

Data were extracted from the patient’s medical records. The datasets used in the current study are available from the corresponding author upon reasonable request.

## List of abbreviations

CT: computed tomography.
NSCLC: non-small cell lung cancer.
VATS: video-assisted thoracoscopic surgery.
